# Telomere length shortening is associated with treatment-free remission in chronic myeloid leukemia patients

**DOI:** 10.1186/s13045-016-0293-y

**Published:** 2016-07-29

**Authors:** Giovanni Caocci, Marianna Greco, Giuseppe Delogu, Christian Secchi, Bruno Martino, Claudia Labate, Elisabetta Abruzzese, Malgorzata Monika Trawinska, Sara Galimberti, Federica Orru, Claudio Fozza, Carlo Gambacorti Passerini, Francesco Galimi, Giorgio La Nasa

**Affiliations:** 1 Hematology Unit, Department of Medical Sciences, Bone Marrow Transplant Center, R. Binaghi Hospital, University of Cagliari, Ospedale “R. Binaghi”, Via Is Guadazzonis, 3, 09126 Cagliari, Italy; 2 Department of Biomedical Sciences, University of Sassari, Sassari, Italy; 3 Istituto Nazionale Biostrutture e Biosistemi, University of Sassari, Sassari, Italy; 4 Division of Hematology, Ospedali Riuniti, Reggio Calabria, Italy; 5 Hematology, S. Eugenio Hospital, University of Tor Vergata, Rome, Italy; 6 Department of Clinical and Experimental Medicine, Section of Hematology, University of Pisa, Pisa, Italy; 7 Department of Clinical and Experimental Medicine, University of Sassari, Sassari, Italy; 8 Department of Internal Medicine, University of Milano Bicocca, Monza, Italy

**Keywords:** Chronic myeloid leukemia, Telomere, Treatment-free remission, Imatinib, Telomerase

## Abstract

We studied telomere length in 32 CML patients who discontinued imatinib after achieving complete molecular remission and 32 age-sex-matched controls. The relative telomere length (RTL) was determined by q-PCR as the telomere to single copy gene (36B4) ratio normalized to a reference sample (K-562 DNA). Age-corrected RTL (acRTL) was also obtained. The 36-month probability of treatment-free remission (TFR) was 59.4 %. TFR patients showed shorter acRTL compared to relapsed (mean ± SD = 0.01 ± 0.14 vs 0.20 ± 0.21; *p* = 0.01). TFR was significantly higher in CML patients with acRTL ≤0.09 (78.9 vs 30.8 %, *p* = 0.002). CML stem cells harboring longer telomeres possibly maintain a proliferative potential after treatment discontinuation.

**Electronic supplementary material:**

The online version of this article (doi:10.1186/s13045-016-0293-y) contains supplementary material, which is available to authorized users.

Telomeres are specialized nucleoprotein structures composed of long arrays of TTAGGG repeats localized at the ends of human chromosomes able to maintain genome stability and integrity and to protect the cell from progressive DNA shortening during repeated division [1]. Telomere biology has been more extensively studied in chronic myeloid leukemia (CML) than in any other blood cancer. Shorter telomeres have been associated with CML, disease progression, poor prognosis, higher Hasford score, and acquisition of cytogenetic aberrations [2–4]. As yet, no studies have considered the possible association between telomere length and treatment-free remission (TFR) after discontinuation of therapy with tyrosine kinase inhibitors (TKIs).

Thirty-two chronic-phase CML patients discontinued TKI treatment after achieving complete molecular remission (CMR) for at least 18 months. All patients received imatinib therapy for more than 24 months. Two patients underwent second-line treatment with nilotinib because of molecular relapse. The median follow-up after discontinuation was 30 months (range 18–60). A complete molecular response was defined as undetectable breakpoint cluster region-Abelson (BCR/ABL1) by real-time quantitative polymerase chain reaction (qRT-PCR) with a sensitivity of the assay corresponding to molecular response (MR)4 and MR4.5. Peripheral blood samples from 32 age- and sex-matched healthy individuals were used for control purposes. The relative telomere length (RTL) was determined by q-PCR according to the technique described by Cawthon in 2002 [5] (Additional file 1). Age-corrected RTL (acRTL) represented the difference in telomere length between patients and age- and sex-matched controls.

The characteristics of 32 chronic phase CML patients are shown in Table 1. Thirteen patients (41 %) showed loss of CMR. All relapsed patients regained CMR after restarting treatment with TKIs. The 36-month cumulative probability of TFR was 59.4 %. RTL was assessed at a mean of 26 months from discontinuation (range 20–30). RTL was assessed at a mean of 26 months from discontinuation (range 18–30). The majority of relapses occurred within 9 months of therapy interruption (mean 8.7 months, range 2–20). In these patients, RTL was assessed after relapse. Overall, median RTL was slightly shorter in patients than in controls (0.97 vs 1.05). The median value of acRTL in the CML cohort was 0.09 (range −0.26, +0.86). The Mann-Whitney *U* test showed shorter acRTL in TFR patients compared to patients with molecular relapse (mean ± SD = 0.01 ± 0.14 vs 0.20 ± 0.21; *p* = 0.01) (Additional file 2). Although the male gender was more frequent in TFR patients, we did not find any significant difference in telomere length between male and female. Patients were stratified according to the median value of acRTL ≤0.09. TFR was significantly higher in CML patients with acRTL ≤0.09 in comparison to those with longer telomeres (78.9 vs 30.8 %, *p* = 0.002) (Fig. 1).

**Table 1:** Characteristics of 32 CML patients according to treatment-free remission (TFR) or molecular relapse after imatinib discontinuation

	Patients in TFR no. 19 (59 %)		Relapsed patients no. 13 (41 %)		*p*
Age at diagnosis (mean, range)	74 (47–88)		56 (37–77)		0.004
Leukocytes at diagnosis ×10^3^/uL (mean, range)	50.47 (8.15–221)		69 (19.8–263)		ns
Platelets at diagnosis ×10^3^/uL (mean, range)	472 (178–918)		357 (171–695)		ns
Months to CMR (median, range)	28 (3–88)		30 (6–93)		ns
Months of TKIs (median, range)	86 (24–127)		84 (45–143)		ns
Months of TKIs >60 (no., %)	13	68.4	12	92.3	ns
Male gender (no., %)	16	84.2	8	61.5	ns
Sokal risk (no., %)					
Low	6	31.6	8	61.5	ns
Intermediate	10	52.6	3	23.1	ns
High	3	15.8	2	15.4	ns
First-line TKI treatment (no., %)	11	57.9	11	84.6	ns
Previous IFN treatment (no., %)	8	42.1	2	15.4	ns
Imatinib first-line TKI treatment (no., %)	19	100	13	100	ns
Nilotinib second-line TKI treatment (no., %)	1	5.3	1	7.7	ns
Age-corrected relative telomere length (mean ± SD)	0.01 ± 0.14		0.20 ± 0.21		0.01

*pres* present, *CMR* complete molecular response, *TKIs* tyrosine kinase inhibitors, *IFN* interferon, *ns* not significant, *SD* standard deviation

**Fig. 1:**
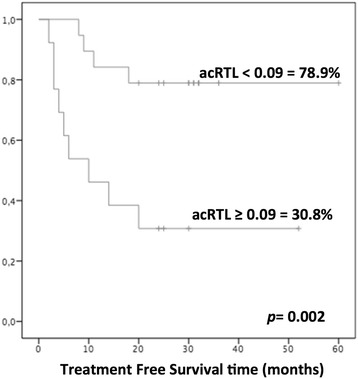
Treatment-free remission (TFR) according to age-corrected relative telomere length (acRTL) ≥0.09 in 32 CML patients

Previous studies suggest a dual-step model for telomere length changes in CML. In the earlier phases, increased turnover of Philadelphia positive (Ph+) progenitors would result in telomere shortening, leading to genetic instability. Later, CML cells would escape senescence and apoptosis through upregulation of telomerase and restored telomere length. This would promote the occurrence of genetically unstable CML subclones with a selective growth advantage [6]. Discontinuation of TKIs is the next hurdle to be overcome in the management of CML patients. Several factors have been identified as potentially capable of predicting durable TFR and hopefully definitive recovery [7, 8]. A significant correlation between younger age and molecular relapse was reported [9]. In our study, CML patients had a slightly shorter telomere length than healthy controls and we found a statistically significant correlation between aging and telomere shortening. However, the most interesting finding was that TFR patients showed significantly shorter acRTL compared to molecular relapses. A possible explanation is that quiescent CML stem cells harboring longer telomeres somehow manage to escape senescence mechanisms and maintain a proliferative potential even after discontinuation of imatinib treatment, but this hypothesis should be supported by CML stem cell telomere assessment in patients with molecular response. Some limitations need to be noted in our study. First, the cohort of patients was relatively small and a longitudinal telomere assessment from diagnosis is lacking. Furthermore, we did not determine sorted myeloid compartment telomere length, but previous reports showed that no significant differences in CML telomere lengths are observed when comparing peripheral mononuclear blood cells, fractionated peripheral neutrophils, and non-fractionated bone marrow mononuclear cells [10]. The present study is the first to suggest that patients with longer telomeres would seem to be more susceptible to relapse after TKI treatment.

## Abbreviations

CML, chronic myeloid leukemia; TFR, treatment-free remission; CMR, complete molecular remission; MR, molecular response; qRT-PCR, real-time quantitative polymerase chain reaction; BCR/ABL1, breakpoint cluster region-Abelson; TKIs, tyrosine kinase inhibitors; RTL, relative telomere length; acRTL, age-corrected relative telomere length; WBC, white blood cell; PLT, platelets

## Additional files

Additional file 1: Additional methods. (DOCX 38 kb) Additional file 2: Boxplot of age-corrected relative telomere length (acRTL) in the group of 19 treatment-free remission (TFR) and 13 molecular-relapsed CML patients. Mean ± SD = 0.01 ± 0.14 vs 0.20 ± 0.21; *p* = 0.01. (TIFF 148 kb)
