# Supplementary material for: Telomere length shortening is associated with treatment-free remission in chronic myeloid leukemia patients
Source: J Hematol Oncol. 2016 Jul 29;9:63. doi: 10.1186/s13045-016-0293-y (PMC4966800; doi:10.1186/s13045-016-0293-y)
Supplement: Additional file 1: — Additional methods. (DOCX 38 kb) [file 13045_2016_293_MOESM1_ESM.docx]

*Supplemental data:*

*Methods*

**Telomere length assessment**

The relative repeat copy number of telomere (T) and a single copy gene (36B4a) (S) were determined by q-PCR according to the technique described by Cawthon in 2002 with a few modifications (5). SYBR Select Master Mix was used for CFX.

Primers tel1b(For) 5′- CGG TTT GTT TGG GTT TGG GTT TGG GTT TGG GTT TGG GTT-3′ (270 nM) and tel2b(Rev) 5′- GGC TTG CCT TAC CCT TAC CCT TAC CCT TAC CCT TAC CCT -3′(900 nM) and primers 36B4 36B4u (For) 5′-CAG CAA GTG GGA AGG TGT AAT CC-3′ (300 nM) and 36B4d (Rev) 5′-CCC ATT CTA TCA TCA ACG GGT ACA A-3′ (500 nM) were used for telomere mixture amplification and gene amplification, respectively. Each sample was analyzed in triplicate. The efficiency of the reaction was determined by means of a standard curve with scalar concentrations of DNA in the calibration sample (20-10-5-2,5-1,25 ng). Amplification was carried out in a Bio-Rad C1000 thermocycler equipped with the CFX96 module. Data obtained from amplification were analyzed by the method of Livak-Schmittgen, also known as a method of 2-ΔΔCT. This method assumes that the amplification efficiency of the target gene (T) and gene reference (S) is close to 100% with a tolerance of ± 5%. (13). The relative telomere length (RTL) was determined as the Telomere (T) to Single copy gene (36B4) (S) ratio (T/S) normalized to a reference sample (K-562 DNA). Age corrected RTL (acRTL) represented the difference in telomere length between patients and age- and sex-matched controls.

***Statistical analysis***

The Kaplan-Meier method was used to calculate the cumulative probability of achieving TFR. The log-rank test compared the groups of patients according to acRTL. The 2 groups of patients either achieving or not achieving TFR were compared using the Mann-Whitney U test or contingency tables, as appropriate. Pearson’s correlation was used to investigate a possible linear association between age and telomere length. Additionally, acRTL, age at diagnosis >45 years, gender, white blood cell (WBC) and platelet (PLT) counts, Sokal risk (low versus intermediate-high), previous interferon treatment, rapidity in obtaining CMR and overall treatment duration >60 months were included in univariate and multivariate analysis. Variables with a p-value lower than 0.2 in univariate analysis were included in multivariate analysis using a multi-step forward binary logistic regression model, where TFR was considered a dependent variable. Only P-values ≤ 0.05 were considered to be statistically significant.
