# Supplementary figures and images for: Telomere length shortening is associated with treatment-free remission in chronic myeloid leukemia patients
Source: J Hematol Oncol. 2016 Jul 29;9:63. doi: 10.1186/s13045-016-0293-y (PMC4966800; doi:10.1186/s13045-016-0293-y)

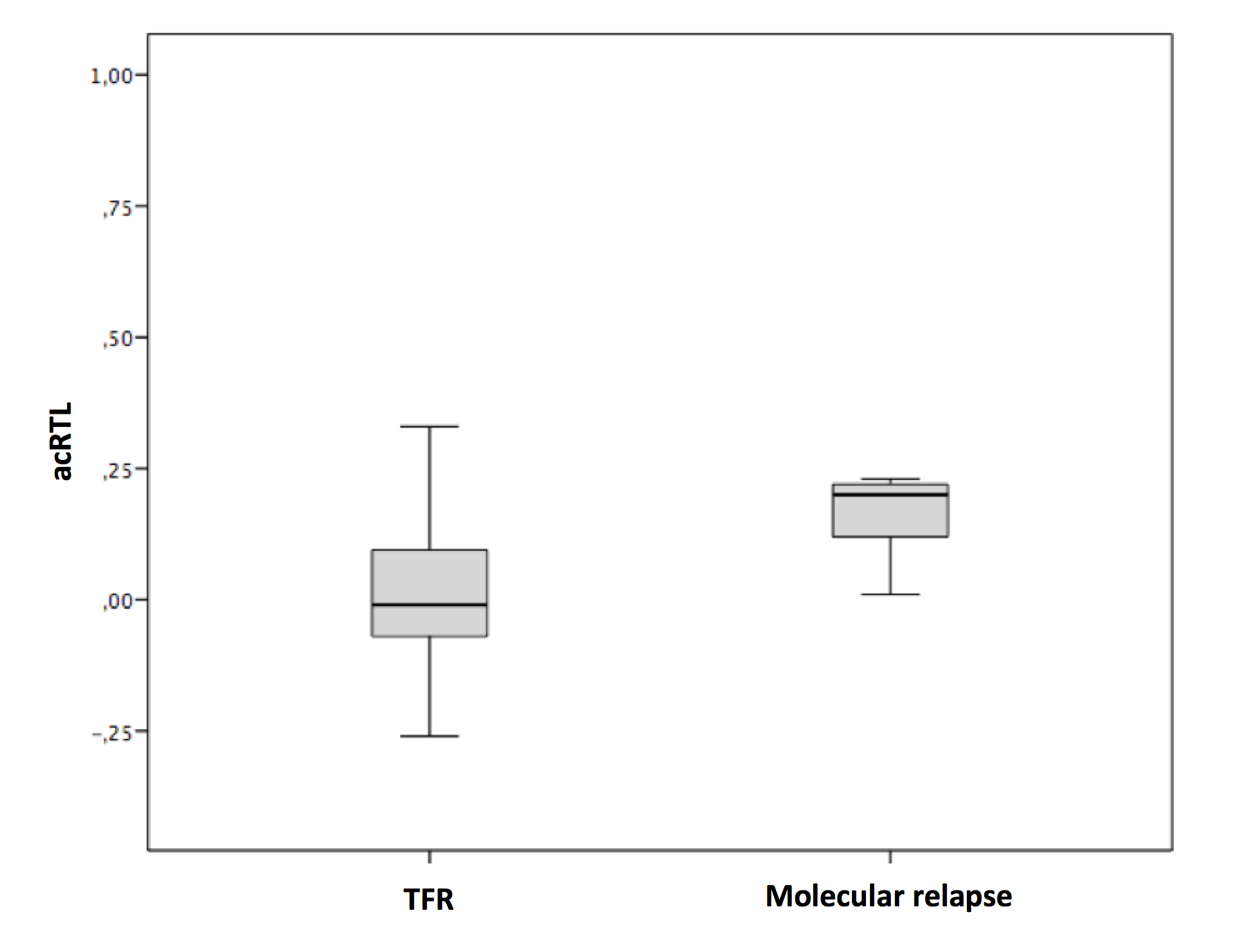

Supplement: Additional file 2: — Boxplot of age-corrected relative telomere length (acRTL) in the group of 19 treatment-free remission (TFR) and 13 molecular-relapsed CML patients. Mean ± SD = 0.01 ± 0.14 vs 0.20 ± 0.21; p = 0.01. (TIFF 148 kb) [file 13045_2016_293_MOESM2_ESM.tiff]
